# Transitory Homicidal Mania: Where Does Reason End or Mania Begin?

**Published:** 1859-10-01

**Authors:** M. le Dr. A. Devergie


### Art. IV.—TRANSITORY HOMICIDAL MANIA: WHERE DOES REASON END OR MANIA BEGIN?

By M. le Dr. A. Devergie.

*(Read before the Imperial Academy of Medicine, Paris.)*

On the 10th of November, 1854, a young man aged nineteen years, the son of one of the principal merchants of Bordeaux, dined with his father, to whom he was much attached, and his stepmother, whom he had regarded from his ninth year first with dislike, and subsequently with gradually increasing aversion.

The dinner, at which several friends should have been present, passed without any unusual incidents. At dessert, young Julius quitted the table, and went to the drawing-room in order to warm himself, but a fire had not been lit there. He then ascended to his chamber and took his fowling-piece and straw-hat, for the purpose of having a stroll in the country, as was his wont, when an idea of suicide, which had tormented him for a month, suddenly occupied his mind, and as suddenly was changed into the thought of *killing his stepmother*.

He threw aside his fowling-piece, went to his brother's room to seek two pistols which had been charged three weeks, although he was ignorant in what manner they were loaded, and at the same time he had pistols of his own that he had charged the day before.

He descended into the dining-room, approached his stepmother, still at table with her husband, and discharged one of the pistols at her temple.

Madame X——— sank down, and the young man recoiling, rested motionless against the wall. His father rose to seize him, but a feeling of self-preservation being aroused in Julius, he fled across the kitchen, through the midst of the terrified domestics, who had run at the sound of fire-arms, and he cried, "*I am a madman—an insensate! I have killed my stepmother!*"

He left the house, surrendered himself to the commissary of police, and related to him the circumstances of the deed.

Before and until this murder the life of this young man had been regular, indeed exemplary; he avoided youths of his own age, or associated but little with them, notwithstanding his great fortune. He fulfilled all the duties of a son and the affection of a brother, and his occupation was regular in a banking-house.

If the deed which the young Julius committed was an act of madness, there must have been a *brusque*, rapid, and instantaneous passage from reason to insanity, and an instantaneous return from insanity to reason. This, then, would be a very clear example of the species of insanity which has been termed transitory.

Where, then, was the limit in this case between reason and madness? Through what grades of change did the intellectual faculties pass to bring about such a transition, and to attain extremes so opposed? This is what we have to ascertain. In the meantime it may be said that the jury of the imperial court at Pau, before which the case was tried, adopted the opinion which MM. Gintrac, Delafosse (of Bordeaux), Calmeil, Tardieu, and myself entertained, and considered the young Julius as not possessing his free-will at the moment of committing the deed, since the court has pronounced a verdict of acquittal, pure and simple.

This judgment is very different from an opinion which was expressed at a period not very far distant, by M. Dupin, at that time advocate, who wrote in these terms to the then prefect of police:—

"Monomania is a new resource of medicine, but it will prove too convenient, at one time to tear the culpable from the just severity of the law; at another, to deprive arbitrarily a citizen of his liberty. When they might not be able to say 'he is guilty,' they will say, 'he is mad,' and Charenton will replace the Bastille."

This was written in the month of March, 1826, in connexion with the case of an individual supposed to be detained unjustly at Charenton. Now, this person had had, from 1804 even to the time of which we speak, a fixed idea that he was loved by all the French princesses. He sent to them, or threw into their carriage, letters, in which he recounted his amorous remembrances. He had been five times under arrest; yet he possessed fully his intellectual faculties upon any other subject, and he was a man of learning; and thus it happened that M. Dupin was led into error.

The afore-mentioned judgment differs, also, very considerably from an opinion given at that time by one of the most eminent magistrates, who said to Marc, in reference to a case similar to that tried before the Imperial Court at Pau:—"*These men are madmen; but it is necessary to cure their mad acts in the Place de Grève.*"

The science of mental alienation has, then, made very great progress, since its doctrines have penetrated even the minds of persons the least acquainted with medicine, and have at once become understood and comprehended by them.

What data has it furnished?

What precepts has it laid down?

These data and these precepts, can they guide the physician in the appreciation of facts, so as to make now evident that which formerly was denied absolutely, since these ideas were at one time rejected entirely by public opinion? It is this that we propose to inquire into; and in order to show how greatly we differ from the past, we shall make an appeal to it.

At the commencement of the present century, Pinel had cast upon the science of mental alienation a light most fruitful for the future. His pupils, Esquirol, Ferrus, and Falret, and the pupils, also, of the last-named men, Georget and Leuret, studied and observed those varieties of insanity which until then had escaped the physicians of that epoch. Marc, following closely upon these so-weighty studies, collected from the annals of justice all the facts which could be grouped around these new ideas.*

Then appeared, in 1825, those remarkable articles of Georget, upon many criminal processes, in the *Archives Générales de Médecine*,† where he assigned and specified the part of each of the intellectual faculties, seeking thus to define them clearly and to establish their respective attributes.

Haste we to say, that Esquirol on the one part, and M. Ferrus on the other, have added lustre to the clear and lucid inquiry of Georget by their learned lectures, their works, and their profoundly elaborated articles in the *Dictionnaire des Sciences Médicales*‡

Then, also, arose those animated discussions between physicians, magistrates, and advocates, upon monomanias; but the acts of transitory madness were but slightly glanced at.

* Marc: *De la Folie, considérée dans ses Rapports avec les Questions Medico-judiciaires.* Paris. 1840. t. i.

† Tomes viii., x., xi., xii., xiii., and xiv.

‡ See Esquirol: *Des Maladies Mentales considérée sous les Rapports Médical, Hygiénique, et Médico-legal.* Paris. 1838. 2 vols. in 8vo.

By a coincidence entirely fortuitous, there had occurred, within a very short space of time, the processes of Leger, Feldtmann, Lecouffe, Jean Poire, Papavoine, and Henriette Cornier, which had opened a wide gate to psychological discussions—discussions which contributed not a little to ensure the prevalence of the principles which had been laid down by the masters of the science.

Thus, in the short period of thirty years or more, we have passed from incredulity, nay more, from the most profound ignorance respecting the varieties of insanity, to an advancement so immense, that at this day magistrates and juries have accepted as clearly established, not only the doctrine of delirious ideas upon a solitary point (*monomania*), but also of transient aberrations of the reason, which, in the eyes of the world, transformed, in times past, the upright man into a criminal, so much the greater that he had carried his perversion of heart even to the extent of hiding during many years, under conduct the most irreproachable, the villany of his disposition.

It is no longer advocates who appeal to science to aid their clients, but magistrates, struck by the enormity of the crime and the feeble interest which has guided the perpetrator of it, address themselves to skilled men, and interrogate them upon the criminality or non-criminality of the deed.

Nevertheless, if monomania, or delirious madness upon a solitary point, with its diversities, its varieties of *haughty*, *homicidal*, *suicidal*, *incendiary*, and *contagious* or *imitative* monomania, are generally accepted by magistrates and juries as implying a fixed delirious, irresistible idea, which fetters the moral liberty, and dominates it entirely, it is necessary to say that it is especially in those cases in which the monomania is accompanied with hallucinations, and of which a fact quoted by Esquirol, among many, affords us a striking example.

A young girl in the Salpêtrière never saw Esquirol approach her without seeking to kill him. Being attacked with sickness, she was placed in the infirmary. One day, preserving the greatest quietude, she suffered Esquirol to approach her, when raising herself hastily upon her bed, she seized him by the cravat in order to strangle him. She was afflicted with homicidal monomania, accompanied with *hallucination*, because she believed that in Esquirol she saw the lover who had deceived her.

Putting aside cases of homicidal monomania, complicated with hallucinations, doubt is still unquestionably entertained by certain magistrates and men of the world, especially when trusting to their own judgment, whatever in other respects may be their capacity and learning. The following fact related by Dr. Rennes, (of Bergerac), will show the grave errors into which magistrates may be led when they do not think it necessary to consult physicians.

B——, a rigorously upright man, loving his mother, and encompassing her with affectionate attentions, married a cousin; but he declared shortly after his marriage, that if he had any children he would desert them. Judge then of the reception which Dr. Rennes received when, at a later date, being called upon to attend the young wife in childbed, he went cheerfully to announce to B. the happy deliverance of the mother! Some time after she was sent back to her family, as well as the infant, which he quickly sought to disinherit.

The mother of B. died. Being ready of hand, and besides very ingenious, this man fabricated a coffin of wood, and one also of lead, and he enclosed in them the corpse of his mother, and shut up the remains in a dark room in his own house. Soon after he believed himself to be surrounded by spies and enemies; and he never went out unless armed to the teeth, when he spread terror around him. Subsequently he thought that it was attempted to poison him; then he bought his provisions himself, prepared his own food, and waited upon himself, and he would not permit any domestic to enter into his house. He collected there powder, lead, guns, and combustibles, ready to defend himself against invasion, and to burn his house, intending to blow out his brains in the midst of the flames.

He sold an estate that was left to him, and the 40,000 francs which he received for it he carried constantly about with him in the crown of his hat, so that nothing might revert to his daughter.

A year passed on, this disordered state of the intellect continuing. All-Saints' day arrived. B. had been over-excited. He met a servant who a year before had been obliged to hide himself, in order to evade the consequences of B.'s vengeance. He fired upon him twice with a fowling-piece, two balls traversing one of the domestic's arms. Another servant who ran to help the wounded one was attacked by B., who cast him down and shattered a leg.

Then the madman entered his house, seized a torch, hastened to the barn of his nearest neighbour, and set fire to it, and afterwards ignited his own house.

Every one hastened to give help, but B. fired eight times successively upon those who approached both the burning buildings. He uttered at each report of his gun strident cries, which resembled the outbursts of an infernal laugh.

Ere long the burning of his own house made progress; an explosion occurred, the accumulation of powder having ignited; and lastly, this furious maniac disappeared in the flames.

And in the midst of the rubbish what remained intact? *Solely the coffin of his mother!*

This was a man in whom the leader of the bar had not been able two months before to recognise the signs of insanity. Indeed, in his calm moments, and beyond the circle of his fixed ideas, B.'s conversation was most consistent. Even his speech possessed a certain charm, and he discussed indifferent subjects wonderfully well. Truly intelligence and sagacity are not sufficient to enable one to judge if a brain be healthy or diseased; it is necessary, in addition, to have studied individuals suffering from every variety and form of insanity.

An immense progress has then been realized by the habitual adoption of medico-legal examinations, in reference to the question of mental alienation, always assumed in doubtful cases.

And if we go back to the species of alienation which forms the subject of this article, transitory insanity, may we not consider that it is a true triumph for science to have been able to obtain the acquittal of the young man of Bordeaux, whose apparently criminal act we have already recorded?

Those physicians who have devoted themselves to the treatment of insanity admit that, besides dementia, mania, and monomania, there exists an instantaneous, transient insanity, which they call *transitory*, and as the result of which an individual, until then, in appearance, at least, of sound mind, commits suddenly an homicidal act, and returns as suddenly to a state of reason.

Seek we, then, to define what ought to be understood by *transitory insanity*. It is not that species of insanity to which Marc and some other physicians have given the name—that is to say, the insanity which shows itself occasionally among epileptic individuals, or among those given to drunkenness; at least we do not understand the term thus. When the *delirious* act is manifested as a sequel of epilepsy or of drunkenness, insane actions precede the criminal deed, and, after its accomplishment, traces of delirium persist for a certain time.

Is that transitory insanity which supervenes as a sequel of persistent emotions, since persistent emotions lead to monomania? The name does not apply here. Murder, committed under the influence of *fanaticism, pride, hate, jealousy, choler*, or *love* has a *known* permanent cause, which acts incessantly upon the moral freedom, and which, in the end, dominates and vanquishes it, bringing about a criminal act.

Violent passions stupify the judgment, but they do not destroy it; they lead the mind to extreme resolves, but they do not deceive it. In a word, the man then acts under the influence of propensities which end by governing, more or less, his actions. But his *conscience* deceives him not. He knows rightly that which he does; he understands the bearing and the consequences. Solely led astray by the passions which have dictated his acts, he trips up his conscience.

Bellart has said that, by assimilating the passions to mental alienation, immorality is justified: it is placed upon the same level as calamity. The man who acts under the empire of passion has commenced by suffering his will to become depraved. The man who acts under the influence of calamity obeys, as a machine, a force, the power of which he cannot contend with.

Finally, it is not well to apply the term *transitory homicidal insanity* to that condition of mind which is developed under the influence of a nature originally depraved, and for which neither *education*, nor *precept*, nor *example*, nor *association*, nor even a *rigid social position* has done anything, but which has been entirely neglected by the individual thus unhappily born, as he falls little by little into infamy.

If, in some of these cases, the motive to action does not justify the action itself, doubt may arise in the mind of the physician; but the criminal act should not then be designated transitory insanity, because it has been gradually induced by social circumstances of an essentially vicious nature. All the causes that we have enumerated, taken singly or in their totality, explain perfectly, in a medical point of view, the delirious idea. Morally and legally speaking, they explain also, up to a certain point, the sudden eruption of an act of delirium; and they would warrant, in certain cases, the admission of attenuating circumstances. But, in addition to insanity developed under the influence of the causes named, it is possible to show another form of alienation to which the term *transitory insanity* ought to be applied—that is to say, a form to the ordinary observer without apparent premonitions, and without appreciable, proximate, or remote cause, manifested as suddenly as the explosion of powder, and ceasing completely with the criminal act. Is this not the history of the young man who has formed the subject of this article, and does not the brief relation which we have made of his reputed criminal act depict sufficiently the species of delirium to which we wish to see attached the denomination *transitory insanity?*

No incentive to the deed, either in passions not sufficiently repressed, or in an acquired fixed idea; antecedents and manners irreproachable; absence of hallucinations; outbreak of insanity manifested by a criminal act, and instantaneous return to reason as soon as the deed was accomplished—these are, according to us, the characters of transitory insanity. Nevertheless, the word *transitory*, perfectly just for the world in general, in the sense that the madness is but transient, though the deed done be of the most criminal description, does not appear to me sufficiently exact for the physician. Individuals of the character described ought not to be considered of sound mind when an idea of crime has suddenly risen within them, when this idea has constituted with them a dominant and irresistible thought, stronger than *the Me*, stronger than the will.

Antecedents of family, divers acts of social life, propensities, tastes more or less perverted, tendencies to taciturnity, ideas of suicide, are often manifested for many years before the explosion of the irresistible criminal idea. So that, to say that *the passage from reason to insanity* can be hasty or instantaneous in the opinion of the physician is to commit an error. This state has prodromata, as every malady has; and, according to us, *if these prodromata do not exist,* it would be impossible to see in the reported criminal act an act of insanity.

Moreover, M. Lelut (*Recherches des Analogies de la Folie et de la Raison,* à la suite de son ouvrage *Le Démon de Socrate,* p. 318) has said, with much truth, in regard to this species of insanity, that at its commencement, and in the mental tendencies which are the predisposing or constitutional cause of it, that insanity is still reason, as reason is already insanity (la folie est encore de la raison, comme la raison est déjà de la folie). This constitutes, for the physician, one of the first elements towards the solution of the question.

A second datum of great interest, in a medical and moral point of view, is the disproportion which exists between the enormity of the offence and the motive or interest which has led to its committal.

If we examine all the criminal processes which have been instituted on the occasion of similar offences, and which have, moreover, been diversely adjudicated upon, but which, for the physician, have been acts of madness, it will be seen that the motive which led to the committal of the deed was not, so far as its consequences were concerned, in relation with the action itself. In other words, the accused, in committing the crime, had in prospect the scaffold; and, even in the case of impunity from it, he derived frequently no advantage, material or moral, from the act which he had committed.

Now, every important act of a man of sound mind has one end. That end is the attainment of an advantage proportionate to the consequences of the act. When an individual stakes his life upon it, he hopes to obtain in exchange material or moral advantages, more or less considerable, and by which he expects to profit largely.

If it be asked what are the conditions under which the reputed criminal act is performed, we are at once struck with the want of foresight which has preceded and accompanied its fulfilment. Neither the moment of the deed nor the means by which it has been effected have been the object of any premeditation. Moreover, the deed has probably been committed at the most unfavourable moment, although the accused had had a thousand opportunities of effecting it in secret.

Far from avoiding justice, the insane individual, in other respects an upright man, comprehending quickly the enormity of the crime that he has involuntarily committed, occasionally—nay, most commonly—gives himself up to justice. In effect, the dominant notion has hastily ceased to exist; moral freedom has resumed its empire, and the so-called criminal has ceased to be mad.

If investigation is extended to the mental state of the paternal or maternal ancestors of the accused, it is common to find that one or more members of the family have committed suicide, or have had a more or less prolonged attack of insanity. Seneca has said:—"Nullum magnum ingenium sine mixturâ dementiæ." Seneca has exaggerated; but Napoleon said truly that "Between a man of genius and a madman there is scarcely the thickness of a six-liards piece." Antiquity presents us in Socrates, Pythagoras, and Democritus, proofs of the exactitude of this assertion, and among men of modern times, Tasso, Pascal, Rousseau still more justify it.

"If I did not fear," says M. Lelut (*Le Démon de Socrate*, Paris, 1856, p. 96) "to renew contemporary griefs, I could show that there are very numerous representatives of art, literature, and science in lunatic asylums. In truth, genius, after having abandoned itself to its highest inspirations, has but one step more to make to break the limit which separates thought from morbid exaltation; the cord too tensely drawn may give way, and then the artist, the poet, the man of science, the philosopher, becomes changed into a poor lunatic: but a moment ago they were the glory of the world, now they are objects of pity." But if we observe those persons who have been attacked with transitory madness, we find generally conditions entirely opposed to these —slight education, little ability, contracted intelligence, and taciturnity, in a word, a monotonous ensemble, both physical and moral.

Lastly (and this is a criterion of great value), if we investigate the offence from two different points of view, the hypothesis of a criminal act, and the hypothesis of an act of folly, in order that either view should be established, it is necessary that it should explain all the facts without effort, while the opposite view should present a series of improbabilities which at once strike the judgment and are inconsistent with experience. This last method leads the physician with the greatest certainty to a right appreciation of the facts; by it doubt is dissipated, conviction arrived at, and the conscience relieved. This method enables us to carry conviction to the minds of magistrates and juries; this, it is necessary to say, brought about the acquittal of young Julius of Bordeaux, and in the manner following. In place of delivering a scientific dissertation upon the question when I was before the court, I avowed that when I had first examined the case I had conceived an unfavourable impression of it; but after I had investigated the offence, as well from the hypothesis of a crime as from the hypothesis of mental alienation, all doubt was dissipated from my mind; and then proceeding in my deposition as I had done in my study, by giving prominence to the past and present state of the accused in their double bearing upon the question of insanity or crime, I was led to the formal conclusion that there had been one of those rapid transitions from the appearances of reason to an act of insanity, which constitutes a species of paroxysm of mental alienation, with its prodromata going back to a remote period and increasing little by little, until the violent outbreak in the reputed criminal act.

When I quitted the witness-box, the Honourable M. Gintrac said to me, "You have saved the accused; from this moment he is acquitted." And, indeed, the next morning the verdict of the jury confirmed the prevision of M. Gintrac. But what did I do more than my four *confrères:* we all concurred in and expressed the same opinion? Nothing, unless it were to reason with the jury as I had reasoned with myself. Yet a few minutes before our examination the Advocate-General had said to me, "Your *confrères* were heard yesterday, and I may tell you that public opinion as well as the opinion of the jury remains unaltered, that is to say, unfavourable to the accused." I do not cite this fact from vanity, but in order that physicians may understand that in doubtful cases, the interpretation of the facts under the double relation I have intimated is one of the elements most fitted to give a solution of the question.

In the case quoted, the young Julius had had among his ancestors a great uncle (maternal) who had a propensity to suicide and who had died insane; an aunt (paternal) who had committed suicide; a third relative (maternal side) who had all his life manifested bizarre and exaggerated ideas, so that it was necessary for him to live a retired life.

On inquiring into the conduct of the young man towards those around him, every one described him as being subject to motiveless outbursts of passion. One day he struck with his hand-whip a servant who was not sufficiently active in attending to his wants; another day he became furiously angry because he could not have immediate access to a room where his stepmother was having a bath. "When he became angry," a witness deposed, "he always seized upon something or some one."

A month before the offence, he had made known to Dr. Brunet his ideas of suicide. He said to the *Juge d'Instruction*, "When I ascended to my room on the day of the offence, I was not thinking of anything. I should not have gone above stairs if I had found a fire in the drawing-room. When I got into my room, having no evil intention, the notion of suicide possessed me; then my thoughts taking another direction, I threw aside my fowling-piece, ran to my brother's chamber, armed myself with two pistols, and re-descended to the drawing-room, *pushed by I know not what force which dragged me in spite of myself*." I would add, also, that in the midst of opulence he enjoyed it not; that he avoided young men of his own age; that he was taciturn, and that he constantly isolated himself.

Finally, he had arrived at that degree of development of the feelings which is neither a healthy nor a morbid state, an organic disposition in virtue of which the well-born man, ambitious of elevated social positions, is led to actions the most sublime; the miserably-born to deeds the most criminal.

If we seek the cause of the offence committed, the motive for the deed, the benefits which the young Julius would derive from it, the preparations for its completion, and the place and moment of its accomplishment, we see nothing but improbabilities, if it be regarded as a criminal act.

So far as *premeditation* and the *choice of arms* are concerned, the accused took his brother's pistols, not knowing in what manner they were charged, although he had loaded his own with ball the day preceding the deed. In respect to the *day on which the crime was accomplished*, it was one on which several friends were expected to dine at the house.

As to the *moment of execution*, it was in the presence of his father, to whom he was much attached, that he killed his stepmother, and such was his veneration for his father that he feared to give him the least pain in the ordinary acts of life. Moreover, he said, during his examination, "If my father had addressed one word to me when I entered the drawing-room, a single word, whatever it might have been, *I should not have killed my stepmother*."

Lastly; it was full day, in the house, in the middle of the domestics, that the deed was committed; and so far from any benefits arising to the homicide by its fulfilment, he had both step-brothers and step-sisters.

Are not all these circumstances unnatural on the hypothesis of a crime, unnatural for the sane man, natural for the madman? But it is said he had conceived a dislike—an aversion even, to his stepmother! This is true; but he had known her since he was nine years of age. He had been surrounded by her cares; those, it is testified, of a fond mother. Did she govern him harshly, or did she control his acts in managing his father's house? Not in the least. Julius, loved by his father, was almost master of the house; not only did he govern his stepmother, notwithstanding his age, but he at times insulted her in presence of the servants. The influence that he exercised was such that he would not suffer his step-brothers and step-sisters to be at his father's table, under the pretext that they made too much noise.

I have entered into these details because I have to justify the principal incident I have cited as an example of transitory homicidal mania. In these cases the part of the physician is every way exceptional. He is not solely consulted upon a legal point, the solution of which would form but a cipher in the balance of justice; it is upon the entire question — the whole process. Magistrates and jury are effaced, so to speak, before the decision which he is called upon to make; the physician pronounces upon the culpability or non-culpability of the accused; by his decision, or with his decision, the crime ceases to be, or the cause proceeds. In face of such a responsibility is it not of weighty import that science should clearly specify the morbid phases which it recognises, and lay down criteria capable of establishing their dominant characters?

This consideration has induced me to define what in my opinion constitutes the characteristics of *transitory insanity* (*folie transitoire*), a vague and elastic expression which ought to be limited to cases analogous to that which I have quoted. And if it be necessary to justify my efforts, we may say that under the term transitory insanity there have been related examples of dementia, mania, and monomania, more or less protracted.

Such was the case of the shoemaker, related by Lævanthal, in Hufeland's *Journal de Médecine*, who, one hour after rising, was seized with incoherence of ideas, and presently, armed with a leather-cutting knife, attacked his wife, who had barely time to escape with her infant. The patient was bled, he became calm, and refreshing sleep followed. Now we may assume that if this man had not been immediately bled he would have become for a longer or shorter time a furious maniac; but this was not a case of transitory insanity.

Was the following instance one of transitory insanity? A day-labourer absented himself from home, begging during two days. On his return he asked for his child. "It is asleep," answered the wife, pointing to a neighbouring closet. The man entered and found there the corpse of his child, which the mother had horribly mutilated, for *it wanted a limb*, which she had *con-*

*verted into food!* When, shortly after, the unhappy lunatic was interrogated by the mayor, she declared that want had constrained her to kill the child, but she had taken care to reserve *the other limbs* for her husband. Was there not unsoundness of mind after as well as before the act of insanity which was termed transitory? And how could that insanity be termed transitory, of which neither the commencement nor the termination was known?

Henrietta C—— was attacked with insanity, not transitory insanity, but *infanticidal* monomania. She had shown a disposition towards the insane act long before its execution, and mental unsoundness remained after its accomplishment; and M. B. de Boismont tells me, that since her trial it has been ascertained that a year before she had been sent to a lunatic asylum for having wished to shorten the days of another infant.

Many other examples related by Marc, Cazauvieth, Heim, and Castelnau, are equally erroneous.

But the farmer whose case is cited by Dr. Edwards (*American Journal of Insanity — Ann. Medico-Psychologiques,* t. IV., 2e. série), was really attacked with transitory insanity. When Dr. Daniel interrogated him upon the cause of his sadness, he answered, "I have undergone a trial which fills me with horror when I think of it. I was laid upon a sofa, and my wife and infants were near the fire. I spoke to them friendlily, when suddenly my eye rested upon the poker. At the same instant I was seized with an idea of shedding their blood. I could not think of anything else. I became wretched; until at last unable to resist any longer, I ordered them, in a voice of thunder, to leave the room. Great God!" added he, "how greatly I thank thee that I am not stained with crime!"

This was transitory insanity, since eight years passed and Dr. Daniel's aid was not again required by the farmer; but it is necessary to add, that this man had passed from an active to an idle life—from poverty to riches; that he had during three years been melancholy and irascible, his aspect being unhealthy; but he had notwithstanding maintained agreeable relations with his neighbours.

There does not exist, then, transitory insanity in the pure acceptation of the term. Transitory insanity, like all other forms of insanity, has its prodromata, its remote and proximate symptoms, which the world apprehends not, and to which it does not attach sufficient importance; and which, sooner or later, explain themselves by the delirious act, the act recognised by every one, often prejudicial, and at times of a criminal character.

And if, with regard to transitory insanity, we ask where reason ends and mental unsoundness commences, although the question cannot be answered, we say that it is necessary first to establish a distinction between the *delirium of insanity* and insanity itself. The explosion of delirium occurs long after the invasion of insanity, and it shows itself in a hasty and sudden manner. As to the insanity itself, it is impossible to lay down the limit which separates it from reason ; it is manifested by successive reasonings and acts, which for the world are acts more or less reasonable or unreasonable, but which, for the physician, are acts more or less imminent of insanity. Still these reasonings and acts are at the commencement so feebly marked that all the sagacity of the physician is necessary in order to appreciate their importance and gravity. In reference to this form of alienation we may reiterate M. Lelut's remark, *that insanity is still reason, as reason is already insanity.*

The individual who has perpetrated a reputed criminal act under the influence of transitory insanity, ought to be regarded as insane after as well as before the deed, notwithstanding the return to reason, because a similar tendency may sooner or later again originate in his diseased mind, and lead to a like result. Hence arises clearly this precept, that the physician of a family cannot too strongly direct the attention of parents to those eccentricities of character and conduct that are too frequently attributed to originality, but which are the beginning of mental derangement. How many outbreaks of insanity would be prevented by a special moral and physical education adapted to each of these cases !

Would not the preventive hygiene of insanity be a grand subject of study ? Certainly, insanity that is not hereditary has its starting points in the primitive organization, education, and social life ; but how great is the number of descendants from idiotic and insane parents, who might be saved from outbursts of insanity by directing their studies, their existence, their social relations and outer life, in such a manner as to fortify the intellectual faculties against all the struggles and contentions of society.

And now, if you please, recall to mind that in 1826, M. Dupin said, that monomania was a new resource of medicine. Remember, also, that in 1833, in this hall, on the occasion of a solemn sitting of the Academy, Marc accumulated fact upon fact, argument upon argument, in order to demonstrate not only that monomania existed, but that it also manifested itself in a *reasoning* form—ratiocinative monomania (*monomanie raisonnante*). If in connexion with these facts we place the recognition even of *transitory insanity*, not only by physicians, but also by magistrates and juries, ought we not to felicitate ourselves upon the immense progress which the science of mental alienation has made in its medico-legal relations ? This progress is due to the persevering efforts of the present generation, of which I should fear to wound the susceptibility if I were to cite names which one day will belong to the history of science. By these persevering efforts many of those social punishments have been put aside, and will still be set aside, which stamp the seal of infamy not only upon the head of an innocent, but also upon his entire family, a diseased brain having been alone in fault.

This result is owing to those physicians of our epoch who have devoted their cares to the insane; to those men whose life has passed in the cold observation of the most cruel of human infirmities, most commonly without the hope of receiving one day from their patients those tokens of gratitude which often reflect greater honour upon the physician than the more ostentatious recompense of fortune.

Towards the end of February I received a letter from the brother of young Julius's victim. Having heard indirectly of the lecture I had delivered before the Academy, he thought it to be his duty to announce to me the death of Julius, and to inform me of the circumstances under which it had taken place. Since 1855, this young man had resided in Brussels. He lived there solitarily. On the 29th of January, he hastily quitted his residence, abandoning his furniture and all that he possessed, and having with him solely his ordinary attire. He went to Bordeaux, and alighted at an hotel, where he passed the night, not visiting either his father or brother. In the morning he purchased a brace of pistols, hired a cab, and was driven to the cemetery, and there at his request he was led to his stepmother's tomb. After sending away his guide, he knelt upon the tomb, wrote several sentences in his debt-book, which he deposited upon the monument, and then blew out his brains. Among the sentences traced in his debt-book there was found the following:—"I wish to die upon the tomb of her whom I have so much loved and regretted!"

How shall we reconcile this assertion, made at the moment of committing suicide, with the opinion entertained by some persons that the cause of the murder was *the deep aversion that the young man had nourished towards his stepmother during ten years?*

Evidently the language as well as the termination of life by suicide are the work of a lunatic. Not the slightest doubt can now be entertained even by the most prejudiced, concerning the correctness of the judgment of the Assize Court at Pau, and the scientific foresight which led to that judgment.

The foregoing information completes an example of transitory insanity which is unique in science, inasmuch as the pathological view of the case has been confirmed by the verdict of a jury.

